# Injury characteristics and their association with clinical complications among emergency care patients in Tanzania

**DOI:** 10.1016/j.afjem.2022.08.001

**Published:** 2022-08-29

**Authors:** Armand Zimmerman, Loren K. Barcenas, Msafiri Pesambili, Francis Sakita, Simon Mallya, Joao Ricardo Nickenig Vissoci, Lawrence Park, Blandina T. Mmbaga, Janet Prvu Bettger, Catherine A. Staton

**Affiliations:** a Duke Global Health Institute, Duke University, Durham, North Carlina, United States; b Department of Emergency Medicine, Duke University Medical Center, Durham, North Carolina, United States; c Kilimanjaro Christian Medical Centre, Moshi, Tanzania; d Kilimanjaro Clinical Research Institute, Moshi, Tanzania

**Keywords:** Injury, Trauma, Emergency medicine, Emergency care, Tanzania, LMICs

## Abstract

**Background:**

Over 5 million people annually die from injuries and millions more sustain non-fatal injuries requiring medical care. Ninety percent of injury deaths occur in low- and middle-income countries (LMICs). This study describes the characteristics, predictors and outcomes of adult acute injury patients presenting to a tertiary referral hospital in a low-income country in sub-Saharan Africa.

**Methods:**

This secondary analysis uses an adult acute injury registry from Kilimanjaro Christian Medical Centre (KCMC) in Moshi, Tanzania. We describe this patient sample in terms of socio-demographics, clinical indicators, injury patterns, treatments, and outcomes at hospital discharge. Outcomes include mortality, length of hospital stay, and functional independence. Associations between patient characteristics and patient outcomes are quantified using Cox proportional hazards models, negative binomial regression, and multivariable logistic regression.

**Results:**

Of all injury patients (n=1365), 39.0% were aged 30 to 49 years and 81.5% were men. Most patients had at least a primary school education (89.6%) and were employed (89.3%). A majority of injuries were road traffic (63.2%), fall (16.8%), or assault (14.0%) related. Self-reported comorbidities included hypertension (5.8%), HIV (3.1%), and diabetes (2.3%). Performed surgeries were classified as orthopedic (32.3%), general (4.1%), neurological (3.7%), or other (59.8%). Most patients reached the hospital at least four hours after injury occurred (53.9%). Mortality was 5.3%, median length of hospital stay was 6.1 days (IQR: 3.1, 15.0), self-care dependence was 54.2%, and locomotion dependence was 41.5%.

**Conclusions:**

Our study sample included primarily young men suffering road traffic crashes with delayed hospital presentations and prolonged hospital stays. Being older, male, and requiring non-orthopedic surgeries or having HIV portends a worse prognosis. Prevention and treatment focused interventions to reduce the burden of injury mortality and morbidity at KCMC are needed to lower injury rates and improve injury outcomes.

## African relevance

• Our patient sample from a national referral hospital serving upwards of 15 million people provides insight into patterns of injury morbidity and mortality in Northwestern Tanzania.
• This study describes the characteristics, predictors and outcomes of adult acute injury patients presenting to a tertiary referral hospital in a low-income country in sub-Saharan Africa.
• Although KCMC receives a large number of injury patients, risk factors for poor outcomes among all-cause injury patients who present to this hospital are not clear.
• Information from this study is intended to aid the improvement of care received by injury patients in the Kilimanjaro region of Tanzania.
• Our findings demonstrate that poor injury outcomes in the Kilimanjaro region may be dependent on injury, clinical, and sociodemographic characteristics.

## Introduction

Injury accounts for 9% of global deaths and 11% of all disability-adjusted life years (DALYs) making it a leading cause of death and disability worldwide [1,2]. Every year nearly 5 million people die from injuries and hundreds of millions more sustain non-fatal injuries that require medical care [3,4]. Non-fatal injuries have been associated with additional behavioral and physical health consequences including violence, substance abuse, and cardiovascular disease [5], [6], [7], [8], [9].

The greatest burden of injury occurs in low and middle-income countries (LMICs) which collectively account for 90% of all injury deaths [1]. Among LMICs, sub-Saharan Africa experiences one of the highest rates of both injury related years lived with disability (YLD) and injury related DALYs [3,4]. While there is clear evidence of a significant injury burden in sub-Saharan Africa, research regarding injury outcomes and their predictors is limited. When comparing high-income countries (HICs) to sub-Saharan LMICs, substantial differences exist in physical access to emergency care services and emergency care quality [10,11]. In addition, sub-Saharan LMIC patients requiring emergency care services may experience considerable in-hospital treatment delays [12], [13], [14]. Given these differences, patient population characteristics and predictors of injury outcomes in HICs may not be generalizable to LMICs in the sub-Saharan region.

Current data from multiple sub-Saharan LMICs suggest common predictors of injury mortality are age, sex, Glasgow Coma Score (GCS), and injury mechanism [15], [16], [17], [18], [19], [20]. Similarly, data from multiple referral hospitals across Kenya suggest common predictors of in-hospital death among all-cause injury patients are age, GCS, injury mechanism, and referral status [15,21]. However, predictors of other outcomes among all-cause injury patients such as length of hospital stay and disability remain predominantly unstudied. Elucidating predictors of poor injury outcomes in sub-Saharan Africa may help inform efforts to improve injury patient care in this region of the world.

Tanzania is a LMIC with a significant injury burden in East sub-Saharan Africa [22,23]. In 2016, the age-standardized incidence of brain and spinal cord injury was 332 cases per 100,000 people. For comparison, the same incidence for HICs in 2016 was 298 cases per 100,000 people [24]. Moshi is a city in the Kilimanjaro region of Northeastern Tanzania. Located within Moshi is Kilimanjaro Christian Medical Centre (KCMC), a tertiary referral hospital serving a population of over 15 million people. Due to its vast catchment area, KCMC treats a large and diverse injury population encompassing brain trauma, spinal cord trauma, abdominal trauma, bone fracture, and burn patients [14,15,[25], [26], [27], [28], [29], [30], [31], [32]].

Although KCMC receives a large number of injury patients, risk factors for poor outcomes among all-cause injury patients who present to this hospital are not clear. Consequently, the objective of this study was to describe the relationships between patient characteristics and in-hospital outcomes among a sample of injury patients presenting to KCMC. Information from this study is intended to aid the improvement of care received by injury patients in the Kilimanjaro region of Tanzania.

## Methods

### Setting

Tanzania is a low-income country in East sub-Saharan Africa with a population of 57 million people. KCMC is one of four national referral hospitals in the country and serves 26% of the total population. The hospital houses 630 patient beds and employs 1300 staff, and the emergency department (ED) receives an average of 80 to 100 patients per day (KCMC website). Upwards of 2000 injury patients may present to KCMC ED annually, with most cases resulting from road traffic crashes [25,33]. Reported mortality estimates range from 11.5% for all-cause injury to 30.0% for brain injury and 35.2% for spinal cord injury [25], [26], [27].

The Tanzanian National Health Insurance Fund (NHIF) is the main provider of health insurance in the country, providing coverage for 7.1% of the population [34]. NHIF members contribute a mandatory 3.0% of their monthly salary to the fund; a contribution that is matched by the government [35]. Services covered by the NHIF include consultations, essential medicines, minor, major and specialized surgeries, inpatient care, rehabilitation, eye care, optics, dental care, and orthopedic equipment. Accounting for other, less resourced insurance schemes, 16.0% of the total population has some form of health insurance coverage [34].

### Participants

We analyzed a registry consisting of adult (≥18 years) patients admitted to KCMC ED for management of acute (<24 hours) injury between April 18, 2018 and May 22, 2019. Patients who were referred to KCMC from another hospital were included in the registry if they were admitted to KCMC within 24 hours of injury occurrence. Our study sample was composed of all patients who consented to registry enrollment.

### Variables

Socio-demographic characteristics included age, sex, education, marital status, tribe, and insurance status. Education was categorized as not completing primary school, completing up to primary school, and completing at least secondary school. Insurance status was categorized as using national health insurance, out of pocket payments, or other. The “other” category included those who left the hospital without paying at all.

Clinical characteristics included HIV status, diabetes status, hypertension status, and alcohol use. HIV, diabetes, and hypertension status were self-reported at the time of admission. Alcohol use was positive if the patient self-reported alcohol consumption up to 6 hours prior to injury, if a clinician determined alcohol intoxication, or if the patient had a positive breathalyzer test on arrival to the hospital.

Injury characteristics included mechanism of injury, severity of injury as measured by the Kampala Trauma Score (KTS), time from injury occurrence to KCMC arrival, whether or not a patient was referred to KCMC from a previous hospital, and type of surgery received. Mechanism of injury was categorized as road traffic incident (RTI), assault, fall, or other (burns, drowning, and unknowns). We dichotomized injury severity as mild and moderate/severe using KTS. KTS is an injury severity score requiring minimal data collection that was developed for use in resource limited settings. The score ranges from 5 (severely injured) to 16 (no serious injuries) and uses information on age, systolic blood pressure, respiratory rate, neurological status, and number of injuries [36]. Since its development, the KTS has been validated in numerous LMIC settings [37]. We classified injury patients as mild if their KTS ranged from 9 to 16 and moderate/severe if it ranged from 0 to 8. All patients who went directly to KCMC after injury occurrence were considered “not referred”’ while those who went to a different hospital first were considered “referred to KCMC”. Type of surgery was categorized as orthopedic (fracture, dislocation), neurological (epidural, spinal), general (laceration, wound, burn), or other (nose, mandible, unknown). Unknown surgeries were those that did not have the type of surgery listed, did not have the reason for surgery listed, or could not be determined based on the patient's chart.

Outcomes included length of stay, mortality, and indicators of self-care and locomotion as measured by the Functional Independence Measure (FIM). Length of stay (LOS) was defined as the number of hospital days from admission to hospital discharge or mortality. Self-care and locomotion were assessed using the FIM. The FIM is a validated tool used to measure physical and cognitive functionality among patients suffering deteriorated locomotion [38]. The FIM contains 18 items graded on a 7-point scale and is broken into two groups: locomotion and cognition. The locomotion subgroup assesses self-care, sphincter control, and locomotion. The cognition subgroup assesses communication and social cognition. For each item a score of 7 indicates complete independence, a score of 5 indicates at least 75% independence, and a score of 1 indicates less than 25% independence [39]. We further broke down the FIM locomotion subgroup into two components: self-care and locomotion. Previous studies have supported this breakdown for the assessment of injury and other rehabilitation patients [40,41]. For each of our two FIM locomotion subgroups we defined a poor outcome as a score less than 5 on any item, as this indicated no degree of functional independence for that item [39].

### Data Analysis

We used descriptive statistics to describe our patient sample. Continuous variables are reported with a median and interquartile range. Categorical variables are reported with frequencies and percentages. Cox proportional-hazards regression was used to estimate hazard ratios and 95% confidence intervals (CIs) for associations between our predictors and mortality. Negative binomial regression was used to estimate rate ratios and 95% CIs for associations between our predictors and LOS (days from admission to the hospital to discharge or death). Multivariable logistic regression was used to estimate odds ratios and 95% CIs for FIM locomotion and FIM self-care. Missing data was imputed using multivariate imputation by chained equations [42]. All analyses were performed using R Software for Statistical Computing [43].

## Results

### Patient Characteristics

A total of 1365 patients were enrolled into the injury registry (Table 1). Our sample was composed primarily of 30- to 49-year-olds (39.0%) and consisted mostly of males (81.5%). A majority of patients were self-employed (36.8%). Other occupations represented included farmers (28.3%), employed skilled workers and professionals (24.2%), and those who were unemployed (10.7%). Most patients (84.5%) accessed care through out-of-pocket payments while only 14.1% of patients were covered by the NHIF.

**Table 1:** Description of Study Sample

Socio-Demographic Characteristics	Age, N (%)	Participants (N = 1365)
	18 to 29 years	528 (38.7)
	30 to 49 years	533 (39.0)
	50 to 64 years	179 (13.1)
	Above 65 years	125 (9.2)
	**Sex, N (%)**	
	Men	1113 (81.5)
	Women	252 (18.5)
	**Education, N (%)**	
	Primary incomplete	142 (10.4)
	Primary complete	719 (52.7)
	Secondary +	504 (36.9)
	**Employment, N (%)**	
	Self-Employed	503 (36.8)
	Farmer	386 (28.3)
	Employed	330 (24.2)
	Unemployed	146 (10.7)
	**Marital Status, N (%)**	
	Married	742 (54.3)
	Single	484 (35.5)
	Other	140 (10.3)
	**Tribe, N (%)**	
	Chagga	714 (52.3)
	Pare	207 (15.2)
	Other	444 (32.5)
	**Insurance Type, N (%)**	
	Out of pocket payment	1154 (84.5)
	National Health Insurance Fund	193 (14.1)
	Other	18 (1.3)
**Clinical Characteristics**	**HIV Status, N (%)**	
	Tested and non-reactive	861 (63.0)
	Never tested	462 (33.8)
	Tested and reactive	42 (3.1)
	**Diabetes, N (%)**	
	Tested and negative	419 (30.7)
	Never tested	914 (67.0)
	Tested and diabetic	32 (2.3)
	**Hypertension, N (%)**	
	Tested and negative	472 (34.6)
	Never tested	814 (59.6)
	Tested and hypertensive	79 (5.8)
	**Alcohol Positive on Arrival, N (%)**	312 (22.9)
**Injury Characteristics**	**Mechanism of Injury, N (%)**	
	Road Traffic Injury	862 (63.2)
	Assault	191 (14.0)
	Fall	229 (16.8)
	Other	83 (6.0)
	**KTS, N (%)**	
	Mild (9-10)	751(55.0)
	Moderate/Severe (0-8)	614 (45.0)
	**Type of Surgery, N (%)**	
	Orthopedic	441(32.3)
	General	56 (4.1)
	Neurological	51 (3.7)
	Other	817 (59.8)
	**Referred, N (%)**	1054 (77.2)
	**Time from Injury to KCMC, N (%)**	
	< 1 hour	168 (12.3)
	1 - 4 hours	461 (33.8)
	> 4 hours	736 (53.9)
**Outcomes**	**In-Hospital Mortality, N (%)**	
	Dead	73 (5.3)
	Alive	1292 (94.7)
	**Length of Stay, Median Days (IQR)**	6.1 (3.1, 15.0)
	**FIM Self-care <5, N (%)**	740 (54.2)
	**FIM Locomotion <5, N (%)**	566 (41.5)

Within our sample, 66.1% of patients had ever received testing for HIV. Forty-two (3.1%) patients had tested reactive for HIV. Few patients had ever received testing for diabetes (33.0%) and only 2.3% of patients were previously diagnosed with diabetes. Only 40.4% of patients had ever been tested for hypertension and 5.8% of patients were previously diagnosed with hypertension. At the time of hospital arrival, 22.9% of patients reported alcohol use up to 6 hours prior to injury occurrence.

The most common mechanism of injury was road traffic incidents (63.2%) followed by falls (16.8%), assault (14.0%), and other causes (6.0%). With regard to injury severity, 55.0% of patients suffered mild injuries while 45.0% suffered moderate/severe injuries. Most surgeries (59.8%) were classified as other, 32.3% as orthopedic, 4.1% as general, and 3.7% as neurological. Overall, a majority of patients took more than four hours (53.9%) to get to KCMC from the time of injury occurrence. Since time from injury occurrence to KCMC arrival depends on a patient's referral status, we performed a sensitivity analysis to determine if an interaction between time from injury occurrence to KCMC arrival and referral status had an effect on our outcomes. Our interaction term between these two variables was insignificant, so we removed the interaction term from our models.

In-hospital mortality was 5.3% and median LOS was 6.1 days (IQR: 3.1, 15.0). In total, 54.2% of patients scored less than 5 on at least one FIM self-care item. Similarly, 41.5% of patients scored less than 5 on at least one FIM locomotion item.

### Associations with Mortality

In our Cox proportional hazards model of mortality (Table 2), patients who did not complete primary school (HR: 1.42; 95% CI: 1.16, 1.74) and those who completed secondary school or higher (HR: 1.21; 95% CI: 1.06, 1.38) had a higher hazard rate in comparison to patients who completed only primary school. Single patients had a higher hazard rate in comparison to married patients (HR: 1.20; 95% CI: 1.03, 1.39). In comparison to patients with road traffic injuries, those who sustained injury through a fall had a higher hazard rate (HR: 1.52; 95% CI: 1.29, 1.80). In comparison to patients making out of pocket payments, those who had some form of insurance other than the NHIF had a lower hazard rate (HR: 0.54; 95% CI: 0.33, 0.92). Patients with moderate/severe injuries had a lower hazard rate in comparison to patients with mild injuries (HR: 0.84; 95% CI: 0.73, 0.96). Patients who received a surgery categorized as “other” had a lower hazard rate in comparison to patients who received orthopedic surgery (HR: 0.83; 95% CI: 0.73, 0.94).

**Table 2:** Associations Between Patient Characteristics and Mortality

	**Alive (N = 826)**	**Dead (N = 68)**	**HR**	**95% CI**
**Age, N (%)**				
18 to 29 years	501 (94.9)	27 (5.1)	Ref	
30 to 49 years	513 (96.2)	20 (3.8)	1.09	(0.94; 1.27)
50 to 64 years	168 (93.9)	11 (6.1)	1.15	(0.92; 1.44)
Above 65 years	110 (88.0)	15 (12.0)	1.25	(0.94; 1.66)
**Sex, N (%)**				
Men	1052 (94.5)	61 (5.5)	Ref	
Women	240 (95.2)	12 (4.8)	0.88	(0.75; 1.03)
**Education, N (%)**				
Primary complete	688 (95.7)	31 (4.3)	Ref	
Primary incomplete	133 (93.7)	9 (6.3)	1.42	(1.16; 1.74)
Secondary +	471 (93.5)	33 (6.5)	1.21	(1.06; 1.38)
**Marital Status**				
Married	698 (94.2)	43 (5.8)	Ref	
Single	462 (95.5)	22 (4.5)	1.20	(1.03; 1.39)
Other	132 (94.3)	8 (5.7)	1.18	(0.98; 1.44)
**Mechanism of Injury, N (%)**				
Road Traffic Injury	819 (95.0)	43 (5.0)	Ref	
Assault	187 (97.9)	4 (2.1)	0.70	(0.59; 0.83)
Fall	210 (91.7)	19 (8.3)	1.52	(1.29; 1.80)
Other	76 (91.6)	7 (8.4)	0.86	(0.68; 1.10)
**Tribe N (%)**				
Chagga	676 (94.7)	38 (5.3)	Ref	
Pare	420 (94.7)	24 (5.3)	0.98	(0.83; 1.15)
Other	196 (94.6)	11 (5.4)	0.92	(0.81; 1.05)
**Employment N (%)**				
Employed	319 (96.7)	11 (3.3)	Ref	
Farmer	360 (93.3)	26 (6.7)	1.11	(0.93; 1.33)
Self-employed	479 (95.2)	24 (4.8)	1.02	(0.88; 1.20)
Unemployed	134 (91.8)	12 (8.2)	0.82	(0.66; 1.01)
**Health Insurance, N (%)**				
Out of pocket payment	1093 (94.7)	61 (5.3)	Ref	
National Health Insurance Fund	182 (94.3)	11 (5.7)	1.03	(0.86; 1.24)
Other	17 (94.4)	1 (5.6)	0.54	(0.33; 0.92)
**KTS, N (%)**				
Mild	740 (98.5)	11 (1.5)	Ref	
Moderate/Severe	552 (89.9)	62 (10.1)	0.84	(0.73; 0.96)
**Type of Surgery, N (%)**				
Orthopedic	435 (98.6)	6 (1.4)	Ref	
Neurologic	40 (78.4)	11 (21.6)	0.76	(0.56; 1.04)
General	53 (94.6)	3 (5.4)	0.87	(0.65; 1.18)
Other	764 (93.5)	53 (6.5)	0.83	(0.73; 0.94)
**HIV, N (%)**				
Tested and non-reactive	828 (96.2)	33 (3.8)	Ref	
Never tested	422 (91.3)	40 (8.7)	0.90	(0.80; 1.03)
Tested and reactive	42 (100)	0 (0)	0.96	(0.70; 1.35)
**Alcohol Status, N (%)**				
Negative	997 (94.7)	56 (5.3)	Ref	
Positive	295 (94.6)	17 (5.4)	0.98	(0.86; 1.13)
**Diabetes, N (%)**				
Tested and negative	392 (93.6)	27 (6.4)	Ref	
Never tested	873 (95.5)	41 (4.5)	1.03	(0.85; 1.26)
Tested and diabetic	27 (84.4)	5 (15.6)	1.24	(0.84; 1.86)
**Hypertension, N (%)**				
Tested and negative	447 (94.7)	25 (5.3)	Ref	
Never tested	775 (95.2)	39 (4.8)	1.06	(0.87; 1.29)
Tested and hypertensive	70 (88.6)	9 (11.4)	1.15	(0.88; 1.52)
**Referred to KCMC, N (%)**				
Not Referred	296 (95.2)	15 (4.8)	Ref	
Referred	996 (94.5)	58 (5.5)	0.97	(0.83; 1.14)
**Time from Injury to KCMC, N (%)**				
< 1 hour	159 (94.6)	9 (5.4)	Ref	
1 - 4 hours	430 (93.3)	31 (6.7)	1.10	(0.90; 1.35)
> 4 hours	703 (95.5)	33 4.5)	1.16	(0.94; 1.44)

### Associations with Length of Stay

In our negative binomial regression model of LOS (Table 3), patients who did not complete primary school (RR: 1.42; 95% CI: 1.16, 1.74) and patients who completed at least secondary school (RR: 1.21; 95% CI: 1.06, 1.38) were more likely to experience longer hospital stays in comparison to patients who only completed primary school. In comparison to married patients, single patients were more likely to experience longer hospital stays (RR: 1.20; 95% CI: 1.03, 1.39). Patients who did not pay out of pocket and who were not covered by the NHIF were less likely to experience longer hospital stays compared to patients who paid out of pocket (RR: 0.54; 95% CI: 0.33, 0.92). With regard to injury mechanism, patients who sustained injury through assault were less likely to experience longer hospital stays compared to those with road traffic injuries (RR: 0.70; 95% CI: 0.59, 0.83). Conversely, patients who sustained injury through falls were more likely to have longer hospital stays compared to those with road traffic injuries (RR: 1.52; 95% CI: 1.29, 1.80). In comparison to patients with mild injuries, those with moderate/severe injuries were less likely to have longer hospital stays (RR: 0.84; 95% CI: 0.73, 0.96). Finally, patients who received an unknown surgery were less likely to experience longer hospital stays in comparison to patients who underwent orthopedic surgery (RR: 0.83; 95% CI: 0.73, 0.94).

### Associations with Disability

In our logistic regression model of FIM self-care (Table 3), patients aged 30 to 49 years old had lower odds of a poor FIM self-care score in comparison to patients aged 18 to 29 years old (OR: 0.35; 95% CI: 0.19, 0.63). In comparison to patients who tested negative for HIV, those who tested positive had higher odds of a poor FIM self-care score (OR: 2.05; 95% CI: 1.05, 4.06). In comparison to patients who did not report alcohol use, those who did had higher odds of a poor FIM self-care score (OR: 1.34; 95% CI: 1.01, 1.77). With regard to injury mechanism, patients who sustained injury through assault (OR: 2.02; 95% CI: 1.43, 2.88) or falls (OR: 1.87; 95% CI: 3.05) had higher odds of a poor FIM self-care score in comparison to patients who suffered road traffic injuries. With regard to surgery, patients who received a general (OR: 3.23; 95% CI: 1.78, 5.93), neurologic (OR: 3.82; 95% CI: 2.06, 7.21), or unknown surgery (OR: 3.47; 95% CI: 2.63, 4.61) had higher odds of a poor FIM self-care score in comparison to patients who received orthopedic surgery. Finally, patients who arrived at the hospital more than four hours after injury occurrence had lower odds of a poor FIM self-care score in comparison to patients who took less than one hour to reach the hospital (OR: 0.46; 95% CI: 0.30, 0.71).

In our logistic regression model of FIM locomotion (Table 3), patients with injuries sustained through assault (OR: 3.63; 95% CI: 2.43, 5.54) or burns/drowning/unknown (OR: 2.18; 95% CI: 1.30, 3.78) had higher odds of a poor FIM locomotion score in comparison to patient who suffered road traffic injuries. In comparison to patients who received orthopedic surgery, those who received general (OR: 3.73; 95% CI: 1.93, 7.60), neurologic (OR: 2.67; 95% CI: 1.41, 5.19), or an unknown surgery (OR: 3.45; 95% CI: 2.62, 4.56) had higher odds of a poor FIM locomotion score. Finally, patients who reached the hospital 1-4 hours (OR: 0.57; 95% CI: 0.37, 0.88) or more than 4 hours (OR: 0.52; 95% CI: 0.33, 0.82) after injury occurrence had lower odds of a poor FIM locomotion score in comparison to patients who reached the hospital within 1 hour.

## Discussion

To our knowledge, this is the first study to investigate risk factors for poor in-hospital outcomes among all-cause injury patients in Tanzania. An understanding of injury epidemiology is a necessary component of improving both injury prevention efforts and trauma care quality in any context. Our patient sample from a national referral hospital serving upwards of 15 million people provides insight into patterns of injury morbidity and mortality in Northwestern Tanzania. Our findings demonstrate that poor injury outcomes in the Kilimanjaro region may be dependent on injury, clinical, and sociodemographic characteristics.

### Injury Characteristics

Consistent with literature from both KCMC and other East sub-Saharan LMICs, RTIs were the most common cause of injury [21,25,[44], [45], [46]]. However, in comparison to RTI patients, those who suffered falls experienced longer hospital stays. Longer hospital stays among fall victims may be explained by age as falls are overrepresented in older aged patients who may require more time to heal or who may have more underlying health complications in comparison to younger patients [21,25,47]. In comparison to patients with RTIs, those who suffered assault, burns, drowning, or other injury mechanisms had higher odds of disability. Higher odds of disability among non RTI patients may be directly attributable to injury severity. For example, burn victims presenting to KCMC have primarily comprised individuals with severe injuries, namely those with second- or third-degree burns [32]. Such patients may be at increased risk of sepsis or contractures, both of which increase the risk of disability and mortality [32]. Alternatively, higher disability odds among non RTI patients may be due to a survivor bias. Severely injured RTI patients are more likely to die in the absence of pre-hospital care [48]. In Tanzania, where a pre-hospital care system does not exist, severely injured RTI patients may die before ever reaching a hospital. Consequently, such patients may not be represented in hospital registries.

Injury patients who received general or neurologic surgeries had higher odds of disability in comparison to patients who received orthopedic surgeries. These associations are consistent with results from a prospective cohort study examining surgical outcomes for 11,422 patients across 25 African countries. The observational study revealed a larger proportion of deaths and complications among abdominal and neurosurgery recipients than in patients receiving orthopedic surgery [49]. At KCMC there is both an orthopedic surgery department and orthopedic rehabilitation unit. However, there are no formally trained neurosurgeons. An absence of specialist surgeons may therefore contribute to higher disability odds among general and neurosurgery recipients. In addition, orthopedic patients have lower chances of a fatal outcome because isolated bony fractures typically result in less severe complications than polytrauma and organ injuries [50,51]. Thus, the hemodynamics of abdominal trauma and head trauma patients is likely the biggest contributor to worse outcomes observed in these patient groups. Lastly, over 60% of African countries lack Emergency Medical Services (EMS) systems [52]. In countries without EMS systems, the absence of prehospital care may exacerbate severe complications resulting from abdominal and head trauma [48].

With regard to time, patients who reached the hospital 1-4 hours or >4 hours after injury occurrence had lower odds of disability compared to patients who reached the hospital within one hour of injury occurrence. However, this is not surprising. First, in the absence of EMS systems patients must be well enough to survive transport to the closest health facility without prehospital care. Thus, it is possible that patients who reach the hospital one or more hours after injury occurrence are those with less severe and therefore more stable injuries [48]. Second, trauma patients who survive one hour without emergency care may be more likely to have better overall outcomes [53]. Finally, in a setting without EMS systems, trauma patients must rely on family, friends, bystanders, or commercial drivers for transport to the hospital [54]. Under these circumstances, the urgency with which a patient is transported to the hospital may depend on the perception of that patient's injury severity by individuals without medical training. Consequently, patients with less severe injuries may be transported at a slower rate than would be expected if such a patient was transported by an ambulance.

### Clinical Characteristics

We found significant associations between HIV comorbidities and poor in-hospital outcomes. In comparison to HIV negative patients, and controlling for KTS, the odds of a poor FIM self-care score was 2.05 times higher among HIV positive patients. An observational study in Rwanda of 504 HIV positive and negative injury patients, matched on KTS and sex, obtained a similar estimate. In comparison to the HIV negative group, the odds of 30-day mortality among those with HIV was 3.60 times higher [55]. Further research regarding the impact of HIV on injury outcomes is necessary, as current literature on the topic is sparse. However, our finding raises an interesting point. In 2017, total government spending on HIV/AIDS and sexually transmitted diseases in Tanzania equated to 5.48% of the country's total health expenditure [56]. For the same year, the government allocated only 0.90% of total health expenditure towards injuries [56]. Despite the effectiveness of expanding surgical services and injury prevention interventions in LMICs, many countries continue to focus investments on communicable diseases [57,58]. Conclusive evidence of an association between HIV and poor injury patient outcomes could support efforts to incorporate HIV testing into trauma care, thereby bridging injury and communicable disease funding.

### Sociodemographic Characteristics

Consistent with global data, males comprised a majority of injury patients in our registry; a trend that has been attributed to differences in engagement with high risk behavior between genders [3]. In our sample, males and adults above 65 years of age trended towards a higher risk of mortality in comparison to females and younger patients respectively. Other studies characterizing injury cases in Tanzania have described the same trend [16,59]. The positive correlation between age and injury mortality has long been established [60]. Given the large percentage of RTIs reported in this study, higher rates of death among males may be explained by gender norms where females are less likely to hold driving related jobs.

With regard to education, patients who completed at least secondary school had a risk of mortality 1.21 times higher than patients who only completed primary school. An observational study including over one thousand patients from four different sub-Saharan LMICs found a similar trend in which the risk of injury among college educated patients was 4.7 times higher than the risk of injury among patients with no formal education [45]. Although our data does not include information on income, we suspect that individuals with higher educational attainment have higher income levels. Furthermore, we hypothesize that individuals with higher income levels have increased access to motor vehicles and are therefore at greater risk of sustaining RTIs.

Interestingly, however, we also found that patients who did not complete primary school had a risk of mortality 1.42 times higher than patients who completed only primary school. Again, although we do not have information on patient income, we suspect patients with lower educational attainment have lower income levels. Such patients may have greater difficulty paying for care as only 16.0% of Tanzania's population is covered by health insurance, leaving 84.0% of the population susceptible to out of pocket payments [34]. Low-income injury patients may therefore be prone to longer hospital stays due to difficulties in obtaining funds necessary to receive surgery or other forms of treatment. Indeed our results indicate longer hospital stays among patients who did not complete primary school. Ultimately, an inability to pay for treatment may increase complications resulting from treatment delays and therefore increase the risk of mortality. We also found longer hospital stays and a higher risk of mortality among single patients in comparison to married patients. We hypothesize that married patients may have a larger social circle of family members on which to source healthcare funding, thus making long hospital stays and death less likely outcomes for this patient demographic.

## Limitations

Several limitations inherent in our study design impact interpretation of our results. First, outcome measures only reflect the status of patients during their hospital stay and do not account for deterioration or improvement in health after leaving the hospital. Second, HIV, diabetes, and hypertension status were self-reported. Social stigma or a lack of knowledge surrounding these conditions may have resulted in prevalence underestimates within our study sample. Third, our study sample only included patients who presented to KCMC ED. Since KCMC is a tertiary hospital, health outcomes at KCMC may be better than health outcomes in less resourced hospitals in the Kilimanjaro region.

## Conclusion

Understanding risk factors for poor outcomes among injury patients is a vital component of reducing the burden of disease in LMICs. This study sought to elucidate such associations among a sample of injury patients presenting to KCMC ED in Moshi, Tanzania. Significant associations were found between age, education, marital status, health insurance type, injury mechanism, injury severity, surgery type, and HIV status with mortality, disability, and length of hospital stay. However, future research investigating injury populations in other regions of Tanzania is required to substantiate these results.

## Dissemination of results

Results from this study, including the present manuscript, will be on display for employees at the Kilimanjaro Christian Medical Centre-Duke Collaboration office. Partial results have been presented at the KCMC clinical case conference, a venue for trainees and professionals at the hospital and university to learn the latest clinical and academic information. These results were also shared in conferences, national and international, with an academic audience, with the hospital staff, and with local stakeholders. Table 3

**Table 3:** Associations Between Patient Characteristics and Length of Hospital Stay/Disability

	**Length of stay RR (95% CI)**	**FIM Self-care < 5 OR (95% CI)**	**FIM Locomotion < 5 OR (95% CI)**
**Age**			
18 to 29 years	Ref		
30 to 49 years	1.09 (0.94; 1.27)	0.35 (0.19; 0.63)	0.83 (0.59; 1.14)
50 to 64 years	1.15 (0.92; 1.44)	0.65 (0.41; 1.02)	0.84 (0.53; 1.35)
Above 65 years	1.25 (0.94; 1.66)	0.57 (0.32; 1.02)	0.68 (0.38; 1.23)
**Sex**			
Men	Ref		
Women	0.88 (0.75; 1.03)	0.90 (0.64; 1.26)	0.80 (0.57; 1.13)
**Education**			
Primary complete	Ref		
Primary incomplete	1.42 (1.16; 1.74)	0.84 (0.54; 1.29)	0.76 (0.49; 1.18)
Secondary +	1.21 (1.06; 1.38)	1.14 (0.87; 1.49)	1.23 (0.93; 1.62)
**Employment**			
Employed	Ref		
Farmer	1.11 (0.93; 1.33)	1.11 (0.78; 1.58)	1.16 (0.81; 1.67)
Self-employed	1.02 (0.88; 1.20)	0.92 (0.67; 1.27)	1.36 (0.98; 1.88)
Unemployed	0.82 (0.66; 1.01)	1.09 (0.71; 1.67)	1.37 (0.88; 2.14)
**Marital Status**			
Married	Ref		
Single	1.20 (1.03; 1.39)	0.97 (0.70; 1.33)	1.03 (0.74; 1.44)
Other	1.18 (0.98; 1.44)	1.60 (1.07; 2.40)	1.16 (0.77; 1.76)
**Tribe**			
Chagga	Ref		
Pare	0.98 (0.83; 1.15)	1.12 (0.79; 1.58)	1.23 (0.88; 1.74)
Other	0.92 (0.81; 1.05)	0.99 (0.76; 1.29)	1.20 (0.92; 1.58)
**Insurance Type**			
Out of pocket payment	Ref		
National Health Insurance Fund	1.03 (0.86; 1.24)	1.05 (0.72; 1.54)	1.03 (0.70; 1.52)
Other	0.54 (0.33; 0.92)	1.92 (0.70; 5.62)	2.44 (0.80; 9.21)
**HIV Status**			
Tested and negative	Ref		
Never tested	0.90 (0.80; 1.03)	1.01 (0.78; 1.30)	0.86 (0.66; 1.11)
HIV positive	0.96 (0.70; 1.35)	2.05 (1.05; 4.06)	1.96 (0.98; 4.11)
**Diabetes**			
Tested and negative	Ref		
Never tested	1.03 (0.85; 1.26)	1.06 (0.71; 1.58)	1.08 (0.71; 1.62)
Positive	1.24 (0.84; 1.86)	1.81 (0.78; 4.23)	1.36 (0.58; 3.24)
**Hypertension**			
Tested and negative	Ref		
Never tested	1.06 (0.87; 1.29)	1.10 (0.74; 1.63)	0.81 (0.54; 1.21)
Positive	1.15 (0.88; 1.52)	0.72 (0.39; 1.28)	1.02 (0.57; 1.82)
**Alcohol Status**			
No	Ref		
Yes	0.98 (0.86; 1.13)	1.34 (1.01; 1.77)	1.30 (0.97; 1.74)
**Mechanism of Injury**			
Road Traffic Injury	Ref		
Assault	0.70 (0.59; 0.83)	2.02 (1.43; 2.88)	3.63 (2.43; 5.54)
Fall	1.52 (1.29; 1.80)	1.13 (0.80; 1.61)	1.19 (0.85; 1.69)
Other	0.86 (0.68; 1.10)	1.87 (1.15; 3.05)	2.18 (1.30; 3.78)
**KTS**			
Mild	Ref		
Moderate/Severe	0.84 (0.73; 0.96)	1.18 (0.90; 1.55)	0.83 (0.62; 1.10)
**Type of Surgery**			
Orthopedic	Ref		
General	0.87 (0.65; 1.18)	3.23 (1.78; 5.93)	3.73 (1.93; 7.60)
Neurologic	0.76 (0.56; 1.04)	3.82 (2.06; 7.21)	2.67 (1.41; 5.19)
Other	0.83 (0.73; 0.94)	3.47 (2.63; 4.61)	3.45 (2.62; 4.56)
**Referred to KCMC**			
Not Referred	Ref		
Referred	0.97 (0.83; 1.14)	1.32 (0.95; 1.83)	1.06 (0.76; 1.49)
**Time from Injury to KCMC**			
< 1 hour	Ref		
1 - 4 hours	1.10 (0.90; 1.35)	0.70 (0.46; 1.06)	0.57 (0.37; 0.88)
> 4 hours	1.16 (0.94; 1.44)	0.46 (0.30; 0.71)	0.52 (0.33; 0.82)

## Authors’ Contribution

Authors contributed as follows to the conception or design of the work; the acquisition, analysis, or interpretation of data for the work; and drafting the work or revising it critically for important intellectual content: AZ contributed 30%; LB 25%; MP 10%; and FS, SM, JRNV, LP, BTM, JPB, and CAS contributed 5% each. All authors approved the version to be published and agreed to be accountable for all aspects of the work.

## Declaration of Competing Interest

The authors declared no conflicts of interest.
